# Moral Judgment as Categorization (MJAC)

**DOI:** 10.1177/1745691621990636

**Published:** 2021-07-15

**Authors:** Cillian McHugh, Marek McGann, Eric R. Igou, Elaine L. Kinsella

**Affiliations:** 1 Department of Psychology, University of Limerick; 2 Social Psychology & Cognition Lab, University of Limerick (SOCOUL); 3 Centre for Social Issues Research, University of Limerick; 4 Department of Psychology, Mary Immaculate College; 5 Health Research Institute, University of Limerick; 6 Research on Influence, Social Networks, & Ethics (RISE) Lab

**Keywords:** morality, categorization, category formation, moral judgments

## Abstract

Observed variability and complexity of judgments of “right” and “wrong” cannot be readily accounted for within extant approaches to understanding moral judgment. In response to this challenge, we present a novel perspective on categorization in moral judgment. Moral judgment as categorization (MJAC) incorporates principles of category formation research while addressing key challenges of existing approaches to moral judgment. People develop skills in making context-relevant categorizations. They learn that various objects (events, behaviors, people, etc.) can be categorized as morally right or wrong. Repetition and rehearsal result in reliable, habitualized categorizations. According to this skill-formation account of moral categorization, the learning and the habitualization of the forming of moral categories occur within goal-directed activity that is sensitive to various contextual influences. By allowing for the complexity of moral judgments, MJAC offers greater explanatory power than existing approaches while also providing opportunities for a diverse range of new research questions.

It’s terribly simple. The good-guys are stalwart and true. The bad-guys are easily distinguished by their pointy horns or black hats and we always defeat them and save the day. Nobody ever dies . . . and everybody lives happily ever after.

—(Whedon, 1997, 41 min, 55 s)

In the above quotation from the TV show *Buffy the Vampire Slayer*, Buffy is being comforted by her mentor Giles. He paints a picture of morality in which good and evil are easily distinguishable. He is, of course, lying. Even in this fantastical world of monsters and demons, there is no consistent, clear way to tell right from wrong.

Morality is a morass, and its complexity poses a considerable challenge to understanding how people make moral judgments. Drawing on moral philosophy, psychologists have adopted labels such as “deontology,” “utilitarianism,” as well as more niche concepts such as “virtue” and “care” to help make sense of people’s varied judgments. Many of the questions about variability or apparent inconsistency in judgments have been understood in terms of people shifting in their implicit (or explicit) moral theories from deontological to utilitarian principles or vice versa. However, attempts to reduce morality to pointy horns or black hats are ultimately doomed to fail, and despite important insights, no theory of moral judgment can yet fully account for the complexity of moral judgment.

In response to these challenges, we present *moral judgment as categorization* (MJAC), which has three premises:

- The making of a moral judgment is a process of categorizing something as *morally right* or *morally wrong* (or indeed *not morally relevant*).
- The process of categorization involved in the making of a moral judgment is a domain-general one (not unique or specific to the moral domain).
- Moral categorization occurs as part of ongoing goal-directed behavior and thus is highly dynamic and sensitive to a range of contextual influences.

We argue that contemporary dynamic approaches to concept and category formation (e.g., Barsalou, 2003, 2017; Barsalou & Wiemer-Hastings, 2005; see also Barrett et al., 2014; Sloman et al., 2008) provide the best framework for making sense of the complexity of moral judgment. MJAC encompasses the same phenomena addressed by extant theories of moral judgment (and phenomena not directly addressed by these theories) but does so in a more parsimonious way. By assuming dynamism and context sensitivity from the outset, MJAC is not subject to ad hoc additions or refinements to cope with variability in moral judgments or to account for less frequently discussed phenomena of moral judgment.

In what follows, we first discuss previous attempts to align moral psychology with the literature on categorization. Second, we present our model, MJAC, in more detail. Third, we assess the strength of evidence for MJAC by contrasting its assumptions, explanations, and predictions with existing moral judgment theories. Fourth, we address specific limitations of MJAC. Finally, we conclude by offering a brief summary of the key advantages and novel research avenues offered by MJAC.

## Moral Judgment and Categorization in the Literature

We propose that research on categorization provides the best understanding of the complexities of moral judgment. Similar arguments have previously been proposed by Stich (1993), Harman et al. (2010), and Prinz (2005); however, these approaches were limited in their ability to account for the full dynamism and context sensitivity of categorization or moral judgments.

Stich (1993) highlighted common examples of moral ambiguity to illustrate that the concepts of right and wrong cannot be defined by a set of necessary and sufficient conditions, thus rejecting the classical view of concepts. However, it is not clear which alternative approach could be adopted in its place. In acknowledging limitations in existing approaches to categorization, Stich argued that developments in both categorization research and morality research should be considered in parallel.

Harman et al. (2010) also rejected the classical view of concepts, proposing that moral judgments can be understood in terms of exemplar models of categorization: “stored representations of instances” of a concept (Harman et al., 2010, p. 234). However, categorizations can be highly variable, sensitive to contextual influences (including sensorimotor, proprioceptive, introspective, and situational influences), and occur as part of goal-directed activity—posing a challenge to exemplar models that assume that categorization is modular, stable, and implicitly taxonomic in organization (for review, see Barsalou, 2003).

Prinz (2005) described the development of dynamic concepts and categories and extended this to the moral domain, representing an important development in the literature. This approach, however, gives more weight to emotions as the source of moral judgments (e.g., “Emotions, I will suggest, are perceptions of our bodily states. To recognize the moral value of an event is, thus, to perceive the perturbation that it causes,” Prinz, 2005, p. 99), whereas the role of the cognitive processes remains unclear. This is particularly challenging in view of recent work advocating a more measured view of the role of emotion in the making of moral judgment (Huebner et al., 2009; Landy & Goodwin, 2015; May, 2014).

Finally, research on categorization processes has also had an indirect influence on theorizing about moral judgment. In presenting and defending the theory of dyadic morality (TDM; Schein & Gray, 2018), the moral domain is described with reference to the broader literature on concepts and categories (e.g., Gray, Waytz, & Young, 2012, p. 206; Gray, Young, & Waytz, 2012, p. 102; Schein & Gray, 2018, p. 42). However, the TDM does not explicitly align with any specific categorization approach. Furthermore, the TDM adopts an essentialist position (see below) that is inconsistent with modern understandings of categorization (Barsalou, 2003; Harman et al., 2010; McCloskey & Glucksberg, 1978; Mervis & Rosch, 1981; Oden, 1977; Rosch, 1975; Rosch & Mervis, 1975; Stich, 1993), and this is problematic for explaining key phenomena in the moral domain.

Essentialism in categorization has been described as a bias to perceive categories as natural kinds or as having an underlying causal structure or “essence” (Gelman, 2003, p. 7). For the purposes of the current discussion, we highlight two types of essentialism. First, we note essentialism in the general population as part of everyday (moral) categorization (for more detailed discussion, see Heiphetz, 2020). Second is essentialism on the part of moral psychologists whereby authors attempt to identify or define a moral essence that distinguishes a moral domain as distinct from other domains, complete with moral-specific psychological processes. We view this as a manifestation of the essentialism error in psychology (see Mesquita et al., 2010). Indeed, the limitations of attempting to identify a moral essence (or necessary and sufficient conditions for something to be moral) have been well documented (Bucciarelli et al., 2008; Knobe, 2018; Sinnott-Armstrong, 2012; Sinnott-Armstrong & Wheatley, 2014; Stich, 1993, 2018). MJAC does not attempt to define a moral essence. Instead, we assume that moral categorization involves domain-general processes.

## Introducing MJAC

The premise of MJAC is that moral judgment is the result of domain-general skill^1^ acquisition in making relevant categorizations in particular instances (Barsalou, 2003, 2017). Consider the formation of the ad hoc goal-derived category *things to pack into a suitcase* (Barsalou, 1991). Items that fall into this category (toothbrush, spare clothes, etc.) are not generally categorized as such on a day-to-day basis. The category emerges as required: when a person needs to pack things into a suitcase. A person who travels frequently will be able to form the category *things to pack into a suitcase* more readily because of repetition and the emerging skill. Barsalou (2003) argued that categorization more generally occurs through the same process.

We propose that this basic process also holds for moral categories; that is, when people encounter a behavior in certain circumstances, they may learn that it is morally wrong, and this behavior becomes associated with the category *morally wrong*. Each subsequent time this behavior is encountered in a context in which its moral value is relevant or it is identified as a member of the category *morally wrong* (either explicitly or implicitly), the person’s skill in deploying this category is strengthened. This same process holds for *morally right*. With the increasing frequency of such categorizations, they become increasingly habitual and automatic (see Barsalou, 2003).

A key strength of the approach adopted here is the ability to account for the dynamism and context dependency (including sensorimotor, proprioceptive, and situational influences) that poses a challenge to other approaches (Barsalou, 2003). One’s interactions with (or exposure to) category members occur as part of goal-directed activity (e.g., general social interactions, gossip, political discussion, setting a “good” example/attempting to appear likable or virtuous, engaging with fiction, jury duty, etc.). This allows for the encountering of an infinite number of categories (e.g., ad hoc goal-derived categories) and category members. Here we are primarily concerned with the superordinate categories of *morally right* and *morally wrong*. These categories display considerable variability. For example, consider the range of emotions associated with the behaviors categorized as *morally wrong*, including anything from murder to stealing office supplies. People may be shocked or angered by murder but might barely show mild contempt in response to stealing paper clips. Similar variability is also observed for the same member of the category depending on the context. Consider (a) lying about the actions of a third party to either cover for them or provide them with an opportunity to come forward themselves, (b) cold-blooded and calculated murder versus killing in a passionate rage, and (c) a “massacre of innocent civilians” versus “collateral damage.”

Despite the variability and context dependency, people’s categorizations show sufficient consistency to give the impression of relative stability. Bananas and apples are reliably categorized as fruit, just as murder and harm are widely categorized as wrong. Barsalou (2003, 2017) provided a framework for the emergence of this relative stability while still accounting for the observed dynamism and context sensitivity.

### Type-token interpretation

Barsalou (1999, 2003) proposed that the learning and maintaining of categorizations occurs through the process of type-token interpretation, defined as the binding of specific tokens (category members) to general types (category). For the category *things to pack into a suitcase* (Barsalou, 1991, 2003), this entails identifying a given item (token) as something that you pack or do not pack into a suitcase (type). Crucially, this process can be implicit, simply involving treating an item as a member or not a member of a particular category within an appropriate context for action, in this case, packing it or not packing it. Skill in forming the categories emerges from repetition and rehearsal of the type-token interpretation; people become skilled at deploying categories that they encounter frequently.

### Context sensitivity

Type-token interpretation occurs every time a given token is encountered such that every categorization of a given token (object/item/event) is subject to contextual influences of the current situation. This results in dynamic and complex categories without necessary and sufficient conditions or even stable best exemplars or prototypes. The properties of an object relevant to that particular context become salient, and the categorization process is accented by the details of the particular circumstances in which the actions are being taken. Stable or recurring properties (both object and contextual) can be learned, and their identification or recognition becomes a part of the subsequent engagement in the relevant goal-directed activity and the enactment of different relevant type-token interpretations of objects. This depends on the experience and learning history of the individual and not inherent in the categories themselves, however, which is what gives rise to the complex, dynamic aspects of concepts central to Barsalou’s approach.

Consider a study by Barsalou (1982). Participants were presented with a series of sentences involving particular items, for example, “The basketball was used when the boat sank” or “The basketball was well worn from much use” (Barsalou, 1982; as described in Barsalou, 2003, p. 537). Following each sentence, participants were asked to verify whether particular properties were true for the item, for example whether “floats” is true for “basketball” after reading either of the above sentences. The fact that basketballs float is relevant to the first sentence, and thus this property is inferred from reading this sentence. In the second sentence, this property (while still true for basketball) is irrelevant and does not become salient by reading the sentence. Thus, although what is true for basketball does not change depending on the situation, the properties that are inferred in a given instance do. This is evident in that participants were faster at verifying floats as true for basketball after reading the first sentence than the second (Barsalou, 1982, 2003). Other studies have yielded similar results and demonstrate that different sentences cause different properties to become salient depending on these properties’ relevance to the given sentence (Greenspan, 1986; Tabossi, 1988; Yeh & Barsalou, 2006). The contextually relevant inferences made when one encounters category members are not limited to object properties but can also include situational and introspective inferences (e.g., Barsalou & Wiemer-Hastings, 2005).

### Habitualization

Highly skilled categorizations become habitualized (automatic/intuitive) to the point that these categorizations provide an illusion of “stable categories.” Typically, these stable categories mirror real-world categories or classes and social norms that are frequently and reliably encountered in day-to-day life. This reflects the use of these categories in (a) interacting effectively with the world and (b) communicating with others. Natural kinds and social norms would constitute prototypical classes of such frequently encountered and reliably implemented categories (e.g., Keil et al., 2004). In some cases, categories that can be referenced to natural kinds may take on the causal rules that distinguish natural kinds. For example, fruit is distinct from vegetables in that the agreed scientific classification of fruit (in our culture) is as containing the seeds. This causal rule is not necessarily operationalized in everyday interactions with fruit and vegetables; however, in certain situations, it may be referenced to aid in the classification of ambiguous items.

More abstract categories are more difficult to define because there may not be a set of causal rules governing membership to draw on. There is a large body of literature documenting the search for causal rules or identifying characteristics of particular emotion categories, for instance, but no approach has fully answered this question (Griffiths, 1997; see also, Barrett et al., 2014; Mesquita et al., 2010).

Barsalou and Wiemer-Hastings (2005) directly addressed this question of abstract concepts and demonstrated that the content of increasingly abstract concepts contains increasingly situational and introspective focus. Consider the possible inferences associated with the categorization of *sofa* compared with *freedom*. Various properties of *sofa* will remain relatively stable across contexts. However, to make sense, any conceptualization of *freedom* needs to be embedded in a specific situational (e.g., freedom from oppression) or introspective (e.g., feeling free) context. Inferences regarding *freedom* are necessarily more context dependent. This results in greater situational or introspective inferences being made for abstract categories, whereas concrete categories allow for more object-level inferences.

The abstract nature of moral categories means they are similarly rich in situational and introspective inferences. That is, whether a particular behavior is viewed as right or wrong varies depending on the situation and may be categorized as right or wrong in different ways specific to the context and the goal-directed activity in which the person is engaged. The link of introspection and the abstract nature of moral categories has been supported by recent approaches that stress the tight coupling of moral judgments and emotions (e.g., Cameron et al., 2013; Huebner et al., 2009; Royzman et al., 2014; Rozin et al., 1999; Valdesolo & DeSteno, 2006).

As with the mapping of habitualized categorizations on to real-world natural kinds, moral categories may appear to follow principles or rules, reflecting social norms of society or a specific social group. A behavior that is encountered frequently and consistently identified as *morally right* may emerge as a “good example” or a *Token*^2^ for *morally right*. Over time, people develop a range of Tokens for the categories *morally right* (and for *morally wrong*). Furthermore, similar behaviors may become categorized together, for example, continued identification of “hitting people” as *wrong* and “kicking people” as *wrong* may lead a person to form the superordinate category *causing harm to people*, which is consistently identified as *wrong*. This may then be taken a step further, and “don’t harm people” and “don’t harm animals” may merge to form *inflicting harm*, which is consistently identified as *wrong*.

The emergence of habitualized, highly generalized, morally grounded Tokens may form the basis of what we call *values*. Furthermore, as more and more Tokens are developed and become increasingly generalized, these generalized Tokens become arranged hierarchically in terms of severity. This essentially becomes one’s moral code. There is not necessarily an underlying set of rules (or moral principles) governing this moral code; it is based on a large collection of Tokens and a process of categorization that is sensitive to context and ongoing actions. Some of the generalized Tokens (values) may appear to exhibit sufficient powers of “governance” to constitute rules. However, these are not true rules; as with the mapping of stable categorizations onto natural kinds, it may be possible to construct plausible (and often true) causes for the associations that define many categories, but the process of categorization remains grounded in type-token interpretation (rather than the rules that can be inferred from referencing observable categories; Barsalou, 2003; Barsalou & Wiemer-Hastings, 2005). MJAC provides a framework for the emergence of what appears to be relative stability in categorization while simultaneously accounting for the observed variability and context dependency that pose a challenge to existing theories of moral judgment.

## Applying MJAC

### Moral dumbfounding

The processes underlying moral judgment, according to MJAC, predict the phenomenon of moral dumbfounding. Moral dumbfounding occurs when people defend a moral judgment even though they cannot provide a reason to support it (Haidt, 2001; Haidt et al., 2000; McHugh et al., 2017). Typically, moral dumbfounding occurs for harmless taboo behaviors (consensual incest, cannibalism involving a body that is already dead). Consider the learning of taboo behaviors as wrong through type-token interpretation and typical interaction with such behavior. The taboo nature of these topics means that they are consistently identified as morally wrong without much discussion (the Scottish public petitions committee notably dismissed a call to legalize incest with no discussion at all; see Sim, 2016). This leads to a high degree of stability in categorizing them as *wrong*. However, although other behaviors may be discussed or disputed, generating a deeper knowledge surrounding the rationale for identifying as right or wrong, the taboo nature of these behaviors prevents them from being discussed. This means that a typical encounter with such behavior involves little more than identifying it as wrong, possibly with an expression of disgust, and changing the subject (Sim, 2016). Identifying causal rules that govern the behavior’s membership of the category *morally wrong* is likely problematic in that a person would have limited experience at attempting to do so. In this view, type-token interpretation of taboo behaviors logically leads to moral dumbfounding.

Phenomena similar to moral dumbfounding have been observed in the nonmoral domain. Although these have not been explicitly identified as “dumbfounding” we suggest that dumbfounding also occurs for categories other than *morally wrong*. For example, Boyd and Keil (Boyd, 1989, 1991; Keil, 1989; see also Griffiths, 1997) found that participants struggled to explain their reasons for categorizing an imagined creature as *a cat* or *not a cat*. Descriptions of participants’ responding in such situations bear a striking similarity whether the target categorization is in the moral domain or not. In discussing their work on the illusion of explanatory depth, Keil et al. (2004) described the sensation of being “surprised by our inability to explain something” (p. 227). Likewise, in discussing moral dumbfounding, Haidt (2001) described how people “express surprise at their inability to find supporting reasons” (p. 817). The illusion of explanatory depth and moral dumbfounding are likely phenomena with common underpinnings.

### Categorizing people versus categorizing actions

In line with Barsalou and Wiemer-Hastings (2005), we have been describing the cognitive processes in relation to the development of the abstract categories *morally wrong* and *morally right*. In reality, people do not deal with these abstractions; rather, moral categorization is situated in specific contexts and occurs as part of goal-directed behavior. In some situations, we may identify specific *actions* as morally questionable or morally praiseworthy, whereas in others, we may identify specific *actors* as morally questionable or morally praiseworthy. Although the *action* or *actor* may belong to the superordinate category *morally wrong* or *morally right* (or *not morally relevant*), it is likely that in everyday interactions, people are more concerned with the subordinate categories in question, for example, *bad/good person* or *bad/good action*.

Authors have argued that when people make moral judgments, the primary evaluation is of the character of the person committing the act (e.g., Landy & Uhlmann, 2018; Uhlmann et al., 2015; see also, Siegel et al., 2017, 2018). MJAC does not adopt this position; rather, we recognize that there are many potential contextual factors that influence whether the target of any given moral categorization is the *actor* or the *action* (or both). The variability relating to the target of moral categorization can influence which superordinate category is eventually implicated, that is, whether the final judgment is *morally wrong* or *morally right* (or *not morally relevant*); for example, if a corrupt politician helps a neighbor with shopping, even though this action may be categorized as good, the actor is likely to still be categorized as bad.

### Moral categorization involving known others

MJAC assumes that moral categorization is dynamic and context dependent. We propose that consideration of the goal-directed nature of moral categorizations provides a key insight into some of the contexts that may affect the target of a given categorization. Consider the following two scenarios:

1. You find out that a colleague has been fired for stealing from your employer—they have been bringing home office equipment for their own personal use, and they have been exaggerating their expense claims.
2. A close friend of yours reveals to you that they have been stealing from their employer—they have been bringing home office equipment for their own personal use, and they have been exaggerating their expense claims.

It seems intuitive that people should judge the second scenario differently from the first scenario, and we predict that people will be more lenient in their judgments of the person in the second scenario than the in first scenario. Despite the historical paucity of research investigating the influence of the relationship between the person making a judgment and the apparent perpetrator (relative to the literature investigating people’s judgments of strangers, see Hester & Gray, 2020; see also Feltz & May, 2017), recent findings support this prediction (Forbes, 2018; Heiphetz & Craig, in press; Hofmann et al., 2014; Lee & Holyoak, 2020; McManus et al., 2020; Weidman et al., 2020). Several studies have demonstrated that people appear to be more lenient in their judgments of people they are close to than they are with strangers (Forbes, 2018; Hofmann et al., 2014; Lee & Holyoak, 2020; Weidman et al., 2020). Further evidence that close others are judged differently to strangers was found by Heiphetz and Craig (in press). They showed that a tendency to dehumanize racists (and sexists) is associated with a greater tendency to view strangers’ ambiguous actions as racially biased (or sexist) but not the ambiguous actions of friends (Heiphetz & Craig, in press). The importance of accounting for possible relationships in moral judgment research is not limited to the relationship between the *observer* and the relevant actors. Recent work has shown that people are judged more favorably for helping strangers than helping kin, whereas a failure to help kin is judged more harshly, suggesting a stronger obligation toward kin than toward strangers (McManus et al., 2020).

A further prediction is that for the second scenario, the target of categorization will be the *action* rather than the *actor*. People are motivated to see close others positively (Forbes, 2018; Murray et al., 1996a, 1996b). If faced with a situation in which a close other committed a moral transgression, people would be motivated to avoid making a negative judgment of the person (Ditto et al., 2009; Murray et al., 1996a, 1996b; Proulx & Inzlicht, 2012). One way to avoid this is to make the target of the categorization the action rather than the actor.^3^

In making the *action* the target of the categorization rather than the *actor*, people can reduce the degree to which they view their close others negatively. However, this strategy is implemented *in addition* to making judgments that are more lenient. Making more lenient judgments about specific transgressions on the basis of the *actor* introduces context-specific inconsistency regarding the categorization of that transgression. MJAC predicts that this inconsistency may threaten the long-term stability of the categorization. Specifically, we predict that leniency toward close others for a specific behavior should eventually lead to more general leniency toward that behavior. This development of more general leniency should occur independently of deliberate attempts to present as consistent (although it could be accelerated by attempts to be consistent), for instance, an increased tolerance of “locker room talk” by people who would otherwise disapprove of sexism.

### Moral categorization involving unknown others

Drawing on the goal-directed nature of moral categorization, MJAC makes a further prediction regarding any *prospective* relationships between the observer and the actor. Effective social interaction involves successfully predicting the actions of others (Waytz & Young, 2018). Thus, a key goal of moral categorization is to distinguish “good” people from “bad” people (Uhlmann et al., 2015) by attempting to identify a person’s moral “essence” (e.g., Dunlea & Heiphetz, 2020) or “character” (e.g., N. Klein & O’Brien, 2016; Siegel et al., 2017, 2018). This enables people to establish relationships or pursue continued interactions with “good” people, and to limit their interactions with “bad” people (or at least treat interactions with “bad” people with caution).

Thus, evaluations of strangers’ actions should show a bias for categorizing the *actor* rather than the *action*. Furthermore, this bias should be more pronounced in situations in which people anticipate that there may be follow-up interactions with the stranger. Research on reciprocity and repeated interactions with strangers or partners (e.g., Fehr & Gächter, 2000, 2003) provides an ideal framework that could be adapted to test this prediction. In conditions in which participants are partnered, their initial evaluations should be more focused on their partner’s character than in conditions in which participants interact with a new “stranger” for each trial.

Drawing on the well-established tendency for negative information to be weighted more heavily than positive information (e.g., Kahneman & Tversky, 1979; Rozin & Royzman, 2001; A. Smith, 1759), we predict that people will be more sensitive to negative actions than positive actions. Indeed, this has been shown to be the case. N. Klein and O’Brien (2016) presented participants with vignettes describing changes in patterns of behavior. Participants were asked to indicate how many consecutive instances of the new behavior would need to occur to convince them that the actor’s “moral character had transformed” (N. Klein & O’Brien, 2016, p. 152). Participants perceived negative transformations much quicker than positive transformations, which was true for commencing negative behaviors and ceasing positive behaviors (N. Klein & O’Brien, 2016). A general heightened sensitivity to negative information means that people appear to be quicker to categorize an actor as “bad” (vs. “good”).

This identification of “bad” actors appears to be present from an early age. Even preverbal infants show a preference for good actors over bad actors (Hamlin et al., 2007, 2010; Hamlin & Wynn, 2011; cf. Margoni & Surian, 2018; Schlingloff et al., 2020; Steckler et al., 2017). We do not claim that infants in these studies have acquired fully developed categories of *morally wrong* and *morally right* and that they assign different actors to these categories. Rather, type-token interpretation predicts that category members should be treated as similar, independently of whether a person can describe the category or even the relationship between the category members.^4^ Previous research has demonstrated that people implicitly treat similar items as similar even though they may not be able to articulate what makes them similar (e.g., recognizing “good decks” from “bad decks” in the Iowa gambling task: Bechara et al., 2005; Damasio, 1994; implicit identification of abstract patterns: Proulx & Heine, 2009; Whitson & Galinsky, 2008).

These findings should not be interpreted as categorizations of “bad” actors being more stable than categorizations of “good” actors. Indeed, the opposite is the case (Siegel et al., 2018), in which beliefs about bad agents are more volatile than beliefs about “good” agents. MJAC explains this volatility in the categorization of “bad” agents relative to “good” agents as emerging because of relative consistency with which categorizations are made. As noted by Siegel et al. (2018), “bad people often behave morally, but good people rarely behave immorally” (p. 750). The contexts in which actors are categorized as “good” are more consistent than the contexts in which they are categorized as “bad.” This consistency makes the categorization “good” actor a more stable categorization than “bad” actor. This apparent stability categorizing “good” actors relative to “bad” actors can also be seen in research on moral essentialism; people show a greater tendency to attribute essence on the basis of moral goodness than moral badness (Heiphetz, 2020; Newman et al., 2014).

The findings discussed above reflect the goal-directed nature of moral categorization. Specifically, people are motivated to understand and predict others’ actions to guide future interactions (Uhlmann et al., 2015; Waytz & Young, 2018). If people understand that some behaviors are associated with positive experiences and some with negative outcomes, then it is not surprising that they show a preference for people who behave in a more positive way, even from a very young age (Hamlin & Wynn, 2011).

Distinguishing between categorizing an *action* or categorizing an *actor* has implications for behavior, specifically when the *actor* in question is the self. In a series of studies by Bryan et al. (2013), participants took part in tasks in which cheating for financial gain (at the expense of the experimenter) was possible. When task instructions discouraging cheating used the term *cheater*, participants’ rates of cheating were significantly lower than when the term used was *cheating*. Committing an action that might fall into the category *morally wrong* is less aversive than being categorized as a *bad person*.

## Examining the Explanatory Power of MJAC

To evaluate the strength of evidence for MJAC, we turn to examine its explanatory power compared with several existing theories of moral judgment. We argue that MJAC ultimately provides greater explanatory power than those models while also keeping the account of the processes involved in moral judgment parsimonious with the currently dominant account of general concept and category formation.

We group the range of extant approaches roughly into two. On the one hand, it may be that variations in moral judgments are a product of variations in the basic functioning of the cognitive system. The expansive range of dual-processing theories has a long history of addressing cognitive variation in such terms. Still, in the case of morality, there are a number of such theories highlighting slightly different forms of a dual-processing distinction. Here we compare MJAC with three theories, each with a slightly different take on the dual-processing view: Greene’s dual-process model (Greene, 2008, 2016; Greene et al., 2001, 2004); more recent, “softer” interpretations of Greene’s approach (Byrd & Conway, 2019; Conway et al., 2018; Conway & Gawronski, 2013; Goldstein-Greenwood et al., 2020); and the model-based/model-free interpretation proposed by both Cushman (2013) and Crockett (2013).

On the other hand, it may be the case that moral judgment arises because of morality-specific processing in which some conditions are met to trigger such morality-focused cognition. MJAC, which follows a dynamic theory of categorization that undermines any form of reliable essence to moral categories, runs counter to this claim. We use the TDM (Gray, Waytz, & Young, 2012; Gray, Young, & Waytz, 2012; Schein & Gray, 2018) as a foil to explore this issue.

A key difference between MJAC and all of the alternative approaches that we identify is that it does not align particular aspects of morality or moral judgment with a single underlying process or processing distinction. On the face of it, this might be seen as complicating matters rather than making sense of them; however, we argue that it has two significant benefits. First, it acknowledges the already clear evidence of complexity and variety when it comes to the making of moral judgments. Second, it makes available a rich set of theoretical resources parsimonious with research in a more general domain of cognitive psychology.

In what follows, we show that the evidence runs against any straightforward mapping between single moral judgment dimensions and underlying cognitive processing. This helps distinguish our account from those already extant in the literature while also providing reasons for seeing moral judgment as being underpinned by more context-sensitive or generic forms of processing.

We then show how what appears to be a wealth of unreliable and inconsistent findings in the domain of moral judgment can be seen to suggest parallels between behavior in moral judgment tasks and well-studied phenomena in categorization research. Although the presence of such parallels only offers suggestive evidence at present, we note that MJAC, at the very least, predicts that such similarities should exist and offers a framework within which systematic relationships between these various phenomena can be sought.

### Beyond unidimensional conceptions of morality

#### Dual-process theories of moral judgment

The three dual-processing theories of moral judgment that we address here each use some form of one-to-one mapping between a key dimension of moral judgment and the underlying differences in information processing expressed in that dual-processing account. Identification of the moral dimension is usually made through categorization of responses to challenges such as the trolley problem (the moral judgment literature is, unfortunately, replete with vehicular homicides).

For instance, Greene’s theory describes the distinction between deontological and consequentialist outcomes to moral judgments as a qualitative difference in processing such that deontological judgments are grounded in implicit, emotional, automatic processing and consequentialist judgments involve deliberate, controlled processing (Greene, 2016). Byrd and Conway’s (2019) softer approach is less dichotomous in that deontological judgments are viewed as involving relatively more affective processing. For both Crockett’s (2013) and Cushman’s (2013) model-free accounts, as opposed to model-based accounts, the logic is similar, although the emphasis is reversed. Whereas for Greene (2016) and Byrd and Conway, the form of processing drives the form of moral judgments, for both Cushman and Crockett, the framing of the moral task drives the kind of processing that is likely to result. Crockett and Cushman both avoided the simple deontological/consequentialist divide but focused instead on evaluating either moral actions or moral outcomes, which give rise to model-free or model-based judgments, respectively. As with Greene and Byrd and Conway, however, they hold a stable one-to-one mapping between this dimension of the content of the moral judgment and the underlying processing.

The clarity of these mappings is appealing, but we argue here that the complexity and inconsistency of the findings in the existing literature on these relationships are disconfirming for these accounts (e.g., De Neys & Białek, 2017; Gamez-Djokic & Molden, 2016; Gubbins & Byrne, 2014; Körner & Volk, 2014; McPhetres et al., 2018; Pizarro & Bloom, 2003; Reynolds & Conway, 2018). Note that research on categorization also predicts reliably distinguishable patterns of response along the lines of many dual-processes accounts, distinguished by individual learning histories and experience in performing given categorizations in different circumstances. For clarity and consistency, we refer to this distinction as one between habitual versus deliberative responses, positioned at either end of a continuum (e.g., Kruglanski & Gigerenzer, 2011).

We follow the categorization research in identifying as a key dimension the extent to which specific categorizations (instances of type-token interpretations) are well rehearsed and thus become fluent, stable, and habitual within frequently enacted goal-directed activities (Barsalou, 1999, 2003, 2017). Less experience with a particular type-token interpretation will result in less consistent deployment of the category and demand more deliberative consideration of the situation and appropriate action.

Therefore, this key dimension in underlying processing is not predicted by MJAC to map straightforwardly onto any aspect of task content or framing in moral judgment, such as habitual judgments being deontological and deliberative ones being consequentialist. Although well-worn deontic exhortations (“It’s wrong to hurt people,” “Thou shalt not kill,” “You shouldn’t hit your sister”) will no doubt develop a strong habitual foundation, within the MJAC framework, consequentialist judgments that are well practiced will also be supported by habitual responses (associated with quick intuitive or affective reactions to moral judgments as studied by De Neys & Białek, 2017; Gubbins & Byrne, 2014; Reynolds & Conway, 2018). Consequentialist reasoning, likely requiring explicit moral argument to arise, may be somewhat less commonly practiced, but also some deontological situations have novel characteristics that therefore also require deliberation (as illustrated by the likes of Gamez-Djokic & Molden, 2016; Körner & Volk, 2014; McPhetres et al., 2018; Pizarro & Bloom, 2003).

This variation in the relationship between deontological and consequentialist judgments and the ways (habitual vs. deliberative) they get made undermines both Greene’s (2016) and Byrd and Conway’s (2019) accounts. Neither Cushman (2013) nor Crockett (2013) connected the moral perspective with a specific form of processing. Still, they did map the distinction between action- and outcome-focused judgments onto the distinction between model-free and model-based processing. Although this can accommodate such variability in deontological or utilitarian perspectives depending on circumstances, it runs afoul of what is termed the *doctrine of double effect* (Doris, 2010; Mikhail, 2000). The doctrine of double effect concerns the difference between causing harm as a means to an end being seen as different to causing harm as a side effect of achieving the same ends even when the actions taken are the same (e.g., Mikhail, 2000; see also R. A. Klein et al., 2018). It is unclear what about such cases could trigger a difference in processing that would explain differential judgments for model theories. These theories are also challenged by versions of the trolley problem presented in virtual-reality environments (Francis et al., 2016), in which a usual pattern of responding (preference for inaction over pushing someone onto the track to stop the tram) was reversed. This runs directly counter to the predictions of the action–outcome mapping to the form of processing made by these model theories. However, the shift to a more deliberative, calculating mode of thinking is perhaps less surprising for MJAC, given the novelty of the mode of presentation.

According to MJAC, the making of moral judgments is dynamic and context dependent and occurs as part of goal-directed activity; thus, we should expect to see this observed variability that poses a challenge to any stable mapping between content and form of processing or judgment outcome. MJAC also assumes that relative stability in moral categorizations emerges as a result of continued and consistent type-token interpretation such that particular categorizations become habitualized (and hence intuitive). Thus, we should expect a variety of contextual factors, not limited to any single key dimension, affecting people’s moral judgments. Constraints on space mitigate against exploring each of these in detail. Still, the sheer range of such factors that have been reported offers compelling evidence that whatever underlies variation in moral judgment is a complex of issues and is not unidimensional in any given situation (the reader is referred to the wealth of literature examining such factors as emotional influences, Cameron et al., 2013; intentionality, evitability, benefit recipient, Christensen et al., 2014; Christensen & Gomila, 2012; action–outcome distinction, Crockett, 2013; Cushman, 2013; trustworthiness and social evaluation, Everett et al., 2016, 2018; personal–impersonal distinction, Greene et al., 2001; doctrine of double effect, Mikhail, 2000; level of physical contact, Valdesolo & DeSteno, 2006; order effects, Wiegmann et al., 2012).

#### Theory of dyadic morality

The TDM (Gray, Young, & Waytz, 2012) that was recently presented by Gray and colleagues would also seem to be grounded in generic categorization processes (Gray, Waytz, & Young, 2012, p. 206; Gray, Young, & Waytz, 2012, p. 102; Schein & Gray, 2018, p. 42). Thus, the approach is not heavily focused on a single processing dimension explaining moral judgment (or the variation therein). Although the TDM has not been identified with a specific theory of categorization, Gray, Waytz, and Young (2012, p. 206) made reference to “prototypes or exemplar sets,” and it is here that the divergence with MJAC becomes clear. Barsalou (2003) summarized a range of findings indicating that neither prototype nor exemplar approaches can adequately explain the dynamic and variable nature of performance in categorization tasks.

More problematically, although TDM has been linked to exemplar and prototype theories, its proponents highlight moral situations as those involving a set of necessary and sufficient conditions—those that involve “an intentional agent causing damage to a vulnerable patient” (Schein & Gray, 2018, p. 33) or “an intentional *moral agent* and a suffering *moral patient*” (Gray, Young, & Waytz, 2012, p. 101). Such appeals to essentialism are at odds with decades of research demonstrating dynamism and context dependency in categorization (Barsalou, 1982, 1987, 1991, 2003, 2017; Harman et al., 2010; McCloskey & Glucksberg, 1978; Mervis & Rosch, 1981; Oden, 1977; Stich, 1993) and returns us to a unidimensional approach to moral judgment, this time identifying the moral character of a situation as the extent to which it involves harm. Although intuitively appealing, this does not bear empirical scrutiny.

Proponents of the TDM argue that even in ostensibly harmless moral transgressions, people perceive harm (Gray et al., 2014). This perception of harm guides participants’ judgments in moral dumbfounding scenarios (Schein, 2020; Schein & Gray, 2018). Dumbfounding is displayed when people maintain a moral judgment in the absence of a means of justifying their judgment, usually evoked by vignettes of supposedly “harmless wrongs” such as consensual incest or cannibalism of an already-dead body (Haidt et al., 2000; McHugh et al., 2017). Schein and Gray (2018) pointed to a series of studies by Royzman et al. (2015) to support their appeal to perceived harm in the moral dumbfounding paradigm. Royzman et al., investigating the case of consensual incest, included additional questions that appear to demonstrate that people’s judgments were (at least in part) grounded in perceptions of harm.

**Fig. 1.**
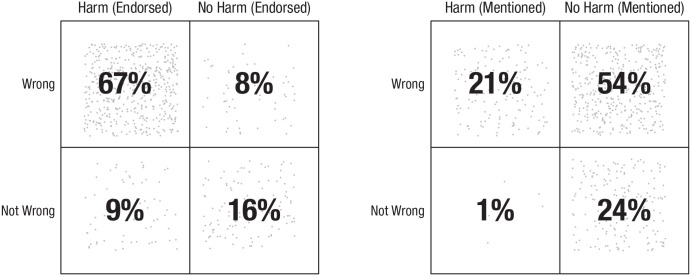
Matrices of combined perceptions of wrongness and perceptions of harm.

However, more recent dumbfounding work fails to support the TDM perspective on this matter (McHugh et al., 2020). In addressing specific methodological limitations of the Royzman et al. (2015) study, McHugh et al. (2020) found that people do not consistently cite harm as a reason for their judgment. Participants were asked to judge a vignette describing consensual incest, asked to provide reasons for their judgment, and then provided with the questions examining perceptions of harm developed by Royzman et al. The responses to the harm-based questions provided one measure of participants’ perceptions of harm, that is, whether participants *endorsed* a harm-based reason for their judgment when it was presented to them. Another measure of perceptions of harm was taken by coding the reasons provided for whether participants *mentioned* harm as justifying their judgment. Figure 1 presents a matrix plotting rows of participants’ judgments (wrong vs. not wrong) against columns of their endorsing of harm (left matrix) or whether they mentioned harm (right matrix) across three studies (*N* = 723).^5^ According to the TDM, all participants should be located in either the top left (harm/wrong) or the bottom right (no harm/not wrong) quadrants. The responding of participants in either of the other two quadrants cannot be explained by the TDM.

Even in taking the most generous measure of perceptions of harm (Fig. 1, left), the responding of 17% of participants (9% + 8%) cannot be explained by the TDM. Taking the stricter (and arguably more accurate, see McHugh et al., 2020) measure of perceptions of harm further reduces the explanatory power of the TDM—only 45% of participants’ responses were in line with the predictions of the TDM.

In addition to evidence for harmless wrongs, the same set of studies had questions explicitly related to the wrongness of behaviors linked with harm and potential harm. Although participants were not explicitly asked about their perceptions of harm for boxing or contact team sports, they were presented with a question: “How would you rate the behavior of two people who engage in an activity that could potentially result in harmful consequences for either of them?” Only 50% of participants across two studies (*N* = 613) rated this as wrong, providing clear evidence for the idea of “wrongless harms” that is rejected by the TDM (Schein & Gray, 2018, p. 43).

So far, there is nothing uniquely “moral” in moral judgment. The people researchers have studied do not appear to apply any given mode of processing or content in a sufficiently consistent manner to provide a stable account of moral judgment. We argue, therefore, that a more successful approach is to explore what the capacity to identify morally right and morally wrong actors, actions, and outcomes has in common with people’s capacity to identify categories more generally.

### Moral phenomena with domain-general (categorization) explanations

MJAC assumes that moral categorization is a dynamic, context-dependent process, and thus, we predict the same phenomena as have been found within the categorization literature at large. In this section, we briefly outline some evidence for this predicted similarity, although we note that at present, these patterns are more suggestive than conclusive. However, we argue that these patterns should be seen not as noise obscuring an underlying stable moral category but as a signal of the complexity of the processes that give rise to that category. We believe that the phenomenon of moral judgment is no more undermined or challenged by this complexity than the cognitive psychology of concepts and category formation are more generally. These include such phenomena as order effects, language effects, the impact of emotions, and typicality of instance.

#### Order effects

In morality research, responses to different moral dilemmas have been found to vary depending on the order of presentation (Petrinovich & O’Neill, 1996; Wiegmann et al., 2012). MJAC can explain these in the same way as order effects in nonmoral categorization are explained. That is, they occur as a result of priming. The scenario that is presented first causes some features of the second scenario to become more salient. The salience of these features leads to a different judgment than if the initial scenario was not presented. In the case of categorization, the effect of this type of priming is primarily studied concerning reaction times. For example, a study by Barsalou (1982, 2003) showed that reading sentences that made particular features of a given object salient influenced the speed at which participants verified related properties of the given object (see also Tabossi, 1988). We predict similar reaction-time variability should be observed when participants are primed with relevant properties for making moral categorizations.

There is also evidence that priming people with particular concepts can influence their subsequent categorizations. In a study by Higgins et al. (1985), participants completed a task in which they were required to create sentences from a selection of words. Some of the words presented were selected to prime a particular concept, for example, *bold*, *courageous*, and *brave* primed *adventurous*, *careless*, and *foolhardy*, respectively, and *rash* primed *reckless* (Higgins et al., 1985, p. 63). Participants were later presented with a description of ambiguous behavior. It was found that the categorizations of these behaviors were influenced by the concept that was primed. A similar study demonstrated the same effect (Srull & Wyer, 1979). We predict that this same effect should occur for moral categorizations, for example, participants’ responses to descriptions of behavior that could be viewed as either “moral” or “self-righteous” or a behavior that could be viewed as either “immoral” or “crafty” should be subject to the same effect as described by Higgins et al. (1985).

#### Language effects

Although the influence of language on the categories available to a given person has a long and controversial history in psychology, recent research has made it increasingly clear that a given language forms a significant constraint on categorization tasks because of the resources of vocabulary and grammatical structure that it provides (Cubelli et al., 2011; Davidoff, 2001). Second-language acquisition also affects how categorizations are formed as a person learns to deploy new linguistic resources in the service of their goal-directed activities (Athanasopoulos, 2007).

People’s moral judgments have been shown to vary depending on whether they read a moral scenario in their first language or in a second language (the “foreign language effect,” e.g., Cipolletti et al., 2016; Costa et al., 2014; Driver, 2020; Geipel et al., 2015; Hayakawa et al., 2017). Specifically, people appear to be more willing to endorse action in the footbridge/push version of the trolley dilemma when this dilemma is presented a language other than their native language. According to MJAC, deontological judgments become intuitive as a result of consistency across contexts. The changing of the language presents a novel context, which means the inferences associated with the *regular* context (e.g., emotional inferences) of encountering or this scenario are not as salient. Evidence for this interpretation comes from research investigating people’s reactions to nonmoral taboo words in their first language compared with a second language. Harris et al. (2003) measured skin conductance of English speakers and Turkish speakers when rating different types of words in their first language and in their second language. It was found that (nonmoral) taboo words led to greater arousal when presented in participants’ first language than when presented in a second language (see also, Colbeck & Bowers, 2012), suggesting that the emotional inferences associated with the footbridge dilemma are less salient when it is presented in a foreign language.

#### Emotion effects

Emotion is perhaps the most widely discussed contextual influence on moral judgments (e.g., Cameron et al., 2013; Giner-Sorolla, 2018; Huebner et al., 2009; Landy & Goodwin, 2015; May, 2014; Prinz, 2005; Royzman et al., 2014; Rozin et al., 1999; Russell & Giner-Sorolla, 2011; Valdesolo & DeSteno, 2006). Above, we outlined how specific emotions may become associated with particular types of judgment, that is, the emergence of relative stability in making specific categorizations is linked with consistency in relevant contextual features in cases in which the relevant contextual features include emotions. In other words, the emotions that may be experienced when a moral categorization is learned (or reinforced/consolidated) are likely to also be present during later categorizations. A corollary of this is that the experience of the specific emotion may provide a contextual cue, reminding people of previous experiences, making a particular categorization more salient (e.g., Barsalou, 2003; Barsalou & Wiemer-Hastings, 2005; Damasio, 1994; Damasio & Damasio, 1994; Rosenfield, 1988).

MJAC predicts that manipulations designed to suppress the salience of these contextual factors (see S. M. Smith & Vela, 2001) should also suppress the influences of emotions on moral categorizations. The foreign-language effect (Colbeck & Bowers, 2012; Costa et al., 2014; Driver, 2020; Geipel et al., 2015; Harris et al., 2003; Hayakawa et al., 2017) described above provides some evidence for this, whereby the salience of the emotional content is reduced by being presented in the second language. Similar effects should be observed using mindset manipulations (Igou, 2011; Igou & Bless, 2007).

The specific contextual influences discussed above provide just a sample of the broader contextual factors known to influence the making of moral judgment. MJAC assumes that moral judgments are dynamic and context dependent, and thus, it is the approach that is best positioned to understand the diverse contextual influences on moral judgment. It is beyond the scope of the current article to describe and account for all the known contextual influences on moral judgment (e.g., an incomplete list would include Bostyn et al., 2018; Christensen et al., 2014; Christensen & Gomila, 2012; Costa et al., 2014; Cushman et al., 2012; Everett et al., 2016, 2018; Forbes, 2018; Francis et al., 2016, 2017; Lee & Holyoak, 2020; Petrinovich & O’Neill, 1996; Rozin et al., 1999; Schein, 2020; Timmons & Byrne, 2019; Uhlmann et al., 2015; Valdesolo & DeSteno, 2006; Vasquez et al., 2001; Vasudev & Hummel, 1987). However, MJAC predicts understanding these diverse context effects depends on (a) accounting for the learning history (e.g., in the cases of emotional influences and the foreign-language effect) and (b) viewing moral categorization as occurring as part of goal-directed activity (e.g., categorization of *actor* vs. *action* discussed above). Incorporating both of these considerations into a program of research inevitably leads to attempts to make the study of moral judgment reflective of real-world moral decision-making (Bauman et al., 2014; Bostyn et al., 2018; Gilligan, 1977, 1993; Hester & Gray, 2020; Hofmann et al., 2014; Schein, 2020; Watkins, 2020).

#### Typicality

Finally, one of the most salient phenomena within the field of categorization concerns the fact that there are “better” and “worse” examples of any given category (McCloskey & Glucksberg, 1978; Oden, 1977). For example, a chair is viewed as a more typical member of the category *furniture* than bookends is (McCloskey & Glucksberg, 1978). Such judgments are made even for those categories with supposedly logical or sharp boundaries, such as geometric figures (Bourne, 1982; Feldman, 2000).

MJAC predicts that this same phenomena of typicality should be observed for moral categorizations, for example, cold-blooded murder versus violence in pursuit of a cause. We further predict that relative typicality should be related to the relative consistency with which category members are identified as members of the given category (and should be independent of perceived severity).

This facet of moral judgment has already seen some discussion in the existing moral judgment theoretical literature. Cushman (2013, p. 282) made a passing reference—that pushing someone “with your hands” is more typically harmful than pushing someone “with your buttocks.” However, typicality sees more substantial discussion in the context of the TDM (Gray & Keeney, 2015; Schein & Gray, 2018).

Typicality ratings in moral judgments, as described by the TDM, are related to the degree to which a given scenario matches the defined prototype of morality, as an “intentional agent causing damage to a vulnerable patient” (Schein & Gray, 2018, p. 32). An act that more clearly involves harm is rated as more typically wrong than an action in which the perceived harm is less. Likewise, if there are evident intentional agents and vulnerable patients, an action is rated as more typically wrong than if the actors are more similar in their intentionality and vulnerability (Gray & Keeney, 2015; Schein & Gray, 2018).

This account of typicality is based on assumptions related to content (agent-patient, harm) and does not inform the understanding of the cognitive processes underlying moral judgments. Thus, it cannot clearly distinguish between typicality and severity. Indeed, the strong overlap between severity of an act and its typicality as an example of moral wrongness is acknowledged: “By definition, more severe acts are more immoral; that is, they are better examples of the category ‘immorality’” (Gray & Keeney, 2015, p. 860).

With MJAC, we propose that typicality is related to both frequency and consistency of exposure, that is, behaviors that are frequently encountered and consistently identified as members of a given moral category should emerge as *typical* category members. Given the consistency with which harm-related transgressions are identified as wrong, the emergence of the prototypical template described by Gray and colleagues is not surprising (Gray & Keeney, 2015; Schein & Gray, 2018). However, we attribute these typicality ratings to the learning history rather than to perceptions of harm and of agents and patients.

Given the possible confounding influence of severity on typicality ratings, unpacking this difference in interpretation will prove challenging; however, we believe it will be a worthwhile endeavor. We hypothesize typicality ratings are related to the learning history and not linked to specific content. This predicts differences in typicality ratings when controlling for severity (either by focusing on harmless dilemmas or by keeping the severity of harm constant). This also predicts differences in typicality ratings within populations via individual differences in moral values (e.g., Graham et al., 2013; Haidt & Joseph, 2008) and between populations via cultural variation (e.g., Haidt et al., 1993). Furthermore, this view of typicality of moral categorizations predicts that perceptions of typicality will be context sensitive, that is, intrapersonal variability should be observed depending on current context and, crucially, on current goal-directed activity. A professor grading papers would rate straight plagiarism as more typically wrong than plagiarism by omitting references. When not grading papers, however, the same professor may be more concerned with the ethics of colleagues’ precarious contracts and entirely indifferent to the shortcuts students may take in their assignments. Similarly, a sports fan may view various behaviors (e.g., overt fouling, cynical fouling, feigning injury so that the referee penalizes the other team) as cheating, or typically wrong when committed by the opposing team, but may turn a blind eye to these same behaviors when committed by members of the team the fan supports.

Note that this sensitivity to context highlights the importance of understanding moral judgments in more real-life contexts rather than through the study of abstract, decontextualized dilemmas (see also Bauman et al., 2014; Bostyn et al., 2018; Gilligan, 1977, 1993; Hester & Gray, 2020; Hofmann et al., 2014; Schein, 2020; Watkins, 2020). By focusing specifically on context-sensitive categorizations occurring as part of goal-directed activity, MJAC offers a framework for attempting to make the study of moral judgments more reflective of the making of moral judgments in everyday life. Furthermore, in recognizing the broader array of contextual influences on moral categorizations, rather than focusing on specific contextual influences on specific types of judgments, MJAC is uniquely positioned to incorporate known context effects into a coherent parsimonious framework. This would provide opportunities for the combined influences of these contextual factors to be studied relative to each other and provide the potential to identify clear boundary conditions to understand how and when specific contextual factors influence moral categorizations more than others.

### Summarizing the differences between MJAC and existing approaches

Above, we outlined how MJAC differs from existing theories in terms of assumptions and explanation. These theories make assumptions on the basis of content, and this results in essentialist theorizing, either implicit or explicit attempts to define an essence of morality. In contrast, MJAC rejects essentialism, instead assuming moral categorizations are dynamic, context dependent, and occurring as part of goal-directed activity. Each of the theories discussed is explicitly or implicitly (e.g., Schein & Gray, 2018, p. 41) based on dual-process assumptions and has related dichotomous assumptions regarding the cognitive mechanisms (where these mechanisms are specified). MJAC does not assume distinct, separable processes but instead adopts type-token interpretation occurring as part of goal-directed activity (Barsalou, 2003, 2017) as the mechanism that underlies moral categorization. These differences in assumptions underlie the differences in the explanation discussed above (for a summary, see Table 1).

**Table 1.** Specific Points of Divergence Between MJAC and Existing Theories

Concept	Greene’s dual-process theory	“Soft” dual-process theory	Model-based accounts	TDM	MJAC
Assumptions					
Content	Deontology–utilitarianism personal–impersonal	Deontology–utilitarianism	Action–outcome	Harm-based, dyadic	Dynamic Context dependent Goal directed
Moral “essence”	(Implicit)	—	(Implicit)	Explicit	Rejected
Processes	Dual processes	Dual processes	Dual processes	(Implicitly dual process)	Continuum
Mechanisms	Intuition (emotion)/cognition	Emotion/cognition	Model-based/model-free	Categorization (unspecified)	Type-token interpretation
Phenomena explained					
Dumbfounding (harmless wrongs)	—	—	Explained	Denied	Explained: learning history
Wrongless harms	—	—	—	Denied	Explained: learning history
Typicality	—	—	—	Matching of “prototype”	Context dependent
Contextual influences	Specific: personal-impersonal	Specific: emotion/cognition	Specific: action-outcome	Specific: harm-based	General: goal-directed activity, learning history

Note: Entries in parentheses are not explicitly articulated. MJAC = moral judgment as categorization; TDM = theory of dyadic morality; — = not discussed.

## Challenges, Limitations, and Responses

MJAC assumes that both relative stability and various contextual influences can be understood in terms of the learning history of the person. Given this assumption, a key challenge associated with MJAC is that it is impossible to gain access to the complete learning history of any person. That said, this limitation is not specific to MJAC; learning influences on moral judgment have been widely discussed (e.g., Campbell, 2017; Crockett, 2013; Cushman, 2013; Haidt, 2003; Kohlberg, 1969, 1985; Narvaez, 2005; Pizarro & Bloom, 2003; Railton, 2017). MJAC proposes making the role of the learning history an explicit consideration in attempting to understand the making of moral judgments. This will be a challenging yet, in our view, worthwhile endeavor, integrating a diverse range of methods and requiring greater interdisciplinary collaboration between the various domains of moral psychology.

Despite predicting a broad range of contextual variability, there remain some influences on moral judgment that are not directly predicted by MJAC. Three such phenomena are the doctrine of double effect, moral luck, and moral conviction. Although not directly predicted, these phenomena further illustrate the variability and complexity that theories of moral judgment must account for.

First, the *doctrine of double effect* is the name given to the finding that people view causing harm as a means to achieving a goal as worse than causing harm as a side effect of achieving a goal (Doris, 2010; Mikhail, 2000). Above, we presented the doctrine of double effect as a limitation of model-based approaches (Crockett, 2013; Cushman, 2013); the action–outcome distinction does not adequately explain why people should make a distinction between harm as a means and harm as a side effect. Likewise, this distinction is not directly predicted by MJAC. It has been found that people apply this distinction even though they cannot reliably articulate it (Cushman et al., 2006; Hauser et al., 2007). This suggests a similarity with moral dumbfounding and the possibility of a common explanation. In the case of moral dumbfounding, MJAC posits that people implicitly learn (through continued and consistent type-token interpretation) that something is wrong and that learning the categorization occurs independently of learning the reasons for the categorization. Distinguishing side effects from means is much more subtle than distinguishing different types of actions; however, there is no reason for such a distinction not to emerge through the same process of type-token interpretation if others are making the same distinction in their moral judgments (Cushman et al., 2006; Hauser et al., 2007; Mikhail, 2000). In this way, although it is not an obvious a priori prediction of MJAC, the doctrine of double effect is not inconsistent with its assumptions.

The second known effect that is not directly predicted by MJAC is the phenomenon of moral luck. Moral luck demonstrates that different outcomes can lead to different evaluations of the *same* behavior (Nagel, 1979, 2013; Williams, 1982; Wolf, 2001; Young et al., 2010). Consider the following two scenarios (adapted from Wolf, 2001; see also Royzman & Kumar, 2004; Williams, 1982):

*Jo*
A truck driver (Jo) needs to make an emergency stop. Jo has neglected to check the brakes of the truck recently. When attempting to stop the truck, Jo loses control, and the truck crashes into the ditch.
*Pat*
A truck driver (Pat) needs to make an emergency stop. Pat has neglected to check the brakes of the truck recently. When attempting to stop the truck, Pat loses control, and the truck runs over a child.

The actions of Jo and Pat are the same, but previous research has shown that in situations like this, people are likely to view Pat as more morally blameworthy than Jo (Walster, 1966; Wells & Gavanski, 1989; Young et al., 2010). People are more harsh in their moral judgments of the same actions when the actions result in negative outcomes. Williams (1982; see Wolf, 2001) is attributed with coining the phrase *moral luck* to describe this asymmetry of judgments of actions based on outcomes.

As with the trolley problem, and the emergence of typicality, MJAC explains the phenomenon of moral luck with reference to the consistency of previous categorizations. Causing harm to another person is relatively consistently categorized as *morally wrong* (Cushman et al., 2012; Schein & Gray, 2018; although not with perfect consistency, e.g., Alicke, 2012; McHugh et al., 2020). This relative consistency means that encountering an event in which the actions of an agent cause harm is highly likely to be categorized as *morally wrong*. The actions described in classic moral luck scenarios are typically ambiguous or minimally problematic. That is, they are not categorized as wrong with the same consistency. This mismatch in the consistency with which the actions as opposed to the outcomes are categorized as wrong leads to what we observe as moral luck. In effect, the harmful outcome may be viewed as a contextual influence that leads to harsher judgments of actions.

A third phenomenon that is not directly addressed by MJAC is moral conviction (e.g., Skitka, 2010), or zeal in moral positions (e.g., McGregor, 2006). Although MJAC does not make specific claims about moral conviction, previous research has linked this to identity and identification with particular groups (e.g., Greene, 2013; see also Heine et al., 2006; Proulx & Inzlicht, 2012), and more recently, attitude strength has been linked with connectivity (e.g., Dalege et al., 2019). We suggest that the meaning maintenance model provides an ideal framework for understanding zeal in moral categorization. According to the meaning maintenance model (Heine et al., 2006), there are four primary domains of meaning: certainty, self-esteem, social relations, and mortality. Nonmoral category knowledge constitutes meaning in the domain of certainty (Heine et al., 2006); moral knowledge additionally holds meaning in the social domain (Greene, 2013; Heine et al., 2006; Proulx & Inzlicht, 2012). We hypothesize that it is this spanning of both the certainty and the social domains of meaning that leads to moral zeal.

When we apply this insight to the broader framework of MJAC, it appears that some contexts (i.e., social/group contexts) matter more in the development of *robust* moral categories. We hypothesize that robustness in moral categorization is related to the consistency of categorization across multiple (social) contexts. Consider the categorization of sexist jokes as *morally wrong*. Some groups would endorse this categorization, and there are groups who would disagree. The degree to which a person will be motivated to defend this categorization will be related to the social groups they are members of and the consistency across these groups. Someone who agrees with this categorization but spends a lot of time tolerating locker room talk will be less zealous than someone who socializes with people who openly identify as feminists.

## Novelties and Conclusion

MJAC builds on the assumption that moral categorization is dynamic and context dependent and occurs as part of goal-directed activity. Given these assumptions, we propose that the best way to understand variability in moral categorization is by accounting for both the learning history and the current goals. Drawing on this, we have identified two general core predictions of MJAC:

- *Stability* in moral categorization emerges through continued and consistent type-token interpretation.
- *Robustness* in moral categorization emerges through consistency across multiple contexts.

In addition to these general core predictions, throughout the preceding discussion, we identified a number of more specific predictions, which are summarized in Table 2. Although some predictions are consistent with existing approaches, other predictions are novel and informed by MJAC.

**Table 2.** Specific Predictions of Moral Judgment as Categorization

Phenomenon	Explanation/general prediction	Specific predictions
Typicality	Continued and consistent type-token interpretation	- Depends on current goals, personal judgments of typicality can vary depending on context/culture
Dumbfounding	Categorization is learned independently of reasons	- Order effects/prior commitments - Inconsistencies readily ignored where possible - Competing goals (consistency vs. principled)
Order effects	Can occur for any category	- Individual differences in categorizations that lead to dumbfounding
	Priming	- Equivalent reaction time effects (e.g., Barsalou, 1982, 2003) - Equivalent flexibility in moral categorization (e.g., Higgins et al., 1985)
Foreign-language effect	Foreign language creates a novel context, reducing influence of contextual influences	- Should be reduced by fluency (but not proficiency), where fluency reflects immersive experience with the language, allowing for the these contextual influences to be reestablished
Emotional influences	Mood-dependent memory	- Mindset manipulations - Drawing attention to possible influence of emotion
Actor/character	We are motivated to view close others positively	- Categorize the action when close other transgresses - Categorize the actor when close other is virtuous
	We are motivated to understand others so we can predict their behavior	- Bias in favor of categorizing actors rather than actions when evaluating the actions of strangers - Especially if there is a possibility for future interactions
Robustness/zeal	Consistency across social contexts leads to more robustness	- People with a more diverse range of social groups should have more tolerance toward alternative views - For any individual, categorizations that are consistent across multiple social groups should be more robust than categorizations that vary between groups

We identified specific phenomena that MJAC can explain better than existing approaches. Furthermore, we identified particular goals that predict specific patterns of variability in the making of moral judgments (e.g., appearing consistent, viewing close others positively, predicting others’ behavior in anticipation of future interactions). We do not present an exhaustive list; however, we illustrate the value of accounting for goal-directed activity in attempting to understand moral categorization.

In addition to the explanatory and predictive power outlined above, a further strength of MJAC is parsimony. If the processes of categorization and making moral judgments have identical underlying cognitive mechanisms, it will be possible to draw on knowledge about the nature of category formation to further the understanding of moral judgments.

It is *not* terribly simple, the good guys are *not* always stalwart and true, and the bad guys are *not* easily distinguished by their pointy horns or black hats. Knowing right from wrong is not a simple process of applying an abstract principle to a particular situation. Decades of research in moral psychology have shown that moral judgments can vary from one situation to the next, and a growing body of evidence indicates that people cannot always provide reasons for their moral judgments. Understanding the making of moral judgments requires accounting for the full complexity and variability of moral judgments. MJAC provides a framework for studying moral judgment that incorporates this dynamism and context dependency into its core assumptions. We argued that this sensitivity to the dynamic and context-dependent nature of moral judgments provides MJAC with superior explanations for known moral phenomena while simultaneously providing MJAC with the power to explain a greater and more diverse range of phenomena than existing approaches.

## Transparency

*Action Editor:* Laura A. King

*Editor:* Laura A. King

## Notes

1. We use the term *skill* to refer to the type of automaticity that is developed through practice/rehearsal. This does not imply any objectivist moral truth that can be accessed by moral “experts.” We refer only to the cognitive processes involved.

2. *Token* denotes a token that may be viewed as prototypical for a given type.

3. Relatedly, for favorable judgments, we predict the opposite effect. That is, if morally praiseworthy actions are being performed by a close other, the target of the categorization is more likely to be the *actor* than the *action*, helping to maintain a positive view of the close other (Forbes, 2018; Murray et al., 1996a, 1996b).

4. Recall the discussion regarding the ad hoc category *things to pack into a suitcase* (Barsalou, 1991, 2003).

5. These figures are not reported in McHugh et al. (2020); however, see McHugh et al. (2018) for full data sets.
